# Supplementary material for: Toward co-design of an AI solution for detection of diarrheal pathogens in drinking water within resource-constrained contexts
Source: PLOS Glob Public Health. 2022 Aug 16;2(8):e0000918. doi: 10.1371/journal.pgph.0000918 (PMC10021207; doi:10.1371/journal.pgph.0000918)
Supplement: Attachment — Submitted filename: Co-design for detection of diarrheal pathogens - Response.pdf [file pgph.0000918.s001.pdf]

EMORY COLLEGE OF ARTS AND SCIENCES
Center for the Study of Human Health

15 June 2022

Dear Dr. Collins Otieno Asweto,

Thank you to you and the reviewers for the thoughtful review of our paper, "Toward co-design of an AI solution for detection of diarrheal pathogens in drinking water within resource-constrained contexts." We believe our revisions and clarifications fully address editorial and reviewer comments.

We have addressed the following editorial comments within the submission portal:

1. Competing interests statements – now complete.
2. Financial disclosure statement – now update to indicate authors funded and role of funder.
3. Data availability – image data have been added on an online DANS repository at 10.17026/dans-2bd-ufvk. Sharing of raw qualitative data is disallowed by our Emory University IRB; in our protocol, we agreed to only report data in aggregate format. The small number of participants and their roles within the partner organization EcoFiltro would be identifiable even with careful standard de-identification procedures.
4. Trademark symbols were removed from the text.
5. Figure files are now uploaded separately in .tif format.
6. We confirm that the photo in Figure 2 was taken by one of our co-authors (Maria Gabriela Croissert Muguercia).
7. We have reviewed the references and have made needed minor revisions to the formatting of citations.

In response to Reviewer 2's helpful comments, we have revised accordingly:

1. *E. coli* as a pathogenic marker of fecal coliforms and indicator of water contamination, and its consequences in causing human diseases of public health concern. – Absolutely a vital point! This has been clarified on page 1 of the manuscript.
2. There is a need to highlight the operational target of the Foldscope and Magic Zoom. For example, Cultural method focuses on isolation and morphological characteristics, Biochemical analysis target the presence of enzymes and metabolites and PCR targets the genes. As an optical machine how does it specifically differentiate E. coli from other coliforms that may be present in the same sample. – This is an important point. We compare our imaging results to gold-standard optical microscopy in which structural morphology is used to identify *E. coli*. We use the AI image processing to compare water sample images to *E. coli* images within the reference database. We have clarified on page 8 of the manuscript.
3. Italicize "E. coli" to "*E. coli*" – Thank you, this has been updated for correct notation throughout the manuscript.

Emory University
550 Asbury Circle
107 Candler Library
Atlanta, Georgia 30322
human.health@emory.edu
humanhealth.emory.edu

EEO/AA/Disability/Veteran Employer

4. How were the participants selected, what consent did they give to participate in co-designing? – Participants were staff at our partner organization EcoFiltro who volunteered to participate. The project was initiated by EcoFiltro as an identified need for their community water quality education efforts. This has been added to page 3 of the manuscript.
5. Reference: Format is inconsistent – We have revised formatting of citations (also editorial comment 7 above).

We attach both a track-changes version and a clean version of the revised manuscript. We are excited to share this proof-of-concept work with the highly interdisciplinary PLOS GPH readership.

We thank you for your consideration.

Sincerely,

Rachel Hall-Clifford

Rachel Hall-Clifford, PhD, MPH, MSc
Assistant Professor, Human Health and Sociology
Emory University
Director, NAPA-OT Field School Guatemala
Co-Founder, safe+natal

103 Tarbutton Hall
1555 Dickey Drive
Atlanta, GA 30322 USA
+1-470-485-1871
